# Giant non-parasitic splenic cyst: a case report

**DOI:** 10.1186/s13256-023-04246-9

**Published:** 2023-12-05

**Authors:** Fereshteh Karbasian, Maryam Ataollahi, Amirali Mashhadiagha, Seyed Ali Moosavi, Mehdi Forooghi, Narges Ansary, Hamed Hosseinian, Bita Geramizadeh

**Affiliations:** 1 Department of Pediatric Gastroenterology, Shiraz University of Medical Sciences, Shiraz, Iran; 2 Shiraz Transplant Research Center, Shiraz University of Medical Sciences٫ , Shiraz, Iran; 3 Student Research Committee, School of Medicine, Shiraz University of Medical Sciences, Zand Blvd., Shiraz, Iran; 4 Department of Pediatric Surgery, Shiraz University of Medical Sciences, Shiraz, Iran

**Keywords:** Splenic cyst, Epidermoid cyst, Hydatid cyst

## Abstract

**Background:**

Splenic cysts are quite rare and asymptomatic. They may result from infection by a parasite, especially *Echinococcus granulosus* (hydatid cyst), or from non-parasitic causes. Since primary splenic cysts are not common, simple cysts can be misdiagnosed with a hydatid cyst in endemic areas.

**Case presentation:**

We reported a 14-year-old Iranian girl initially presented with a vague abdominal pain, which progressed to left shoulder pain, fullness, early satiety, and shortness of breath and remained undiagnosed for 7 months despite seeking medical attention. Finally, imaging revealed a massive splenic cyst measuring 220 mm × 150 mm × 160 mm raising concern for a hydatid cyst due to regional endemicity. Consequently, the patient underwent total splenectomy. However, histopathological examination surprisingly revealed a simple non-parasitic cyst.

**Conclusions:**

Detecting rare simple spleen cysts requires early ultrasonography (US) and careful reassessment of diagnoses for non-responsive or worsening symptoms. Distinguishing them from splenic hydatidosis, especially in endemic areas, demands thorough paraclinical evaluations and patient history regarding potential parasitic exposure. While total splenectomy is the primary treatment for these huge cysts, the optimal surgical approach should be tailored case by case. These insights emphasize a comprehensive diagnostic approach to enhance accuracy and optimize patient care for these uncommon cysts.

## Introduction

Simple splenic cysts, although rare with an estimated incidence of approximately 0.75 per 100,000 of the population, pose a notable area of interest within the literature. Often asymptomatic and incidentally discovered through imaging studies, understanding the diverse etiologies, classifications, and appropriate treatment modalities for splenic cysts is crucial for informed medical practice [1].

They can be due to parasitic causes, mainly the parasite *Echinococcus granulosus* (hydatid cyst), or non-parasitic causes. Non-parasitic cysts are categorized as primary, lined by an epithelial cover (epidermoid, dermoid, and mesothelial) or endothelial cover (hemangioma, lymphangioma), and secondary (pseudocysts, non-epithelial), which usually have a post-traumatic origin [2, 3].

Total or partial splenectomy is the accepted treatment; however, parasitic infections must be excluded preceding operation [4]. Still, improved perception of the significant immunological role of the spleen has led to alternative spleen-preserving options. Therefore, a laparoscopic method may be preferable to an open procedure due to shorter hospital admission and less postoperative pain [5].

Limited number of similar cases have been reported previously, which are provided in the discussion, but we aimed to present this case of huge non-parasitic splenic cysts due to its exceptional educational path of lengthened diagnosis, challenging operation, and game-changing pathologic study. This case contributes to the medical literature by offering a real-world example that supports and reinforces the existing knowledge regarding the diagnosis, classification, and appropriate management of splenic cysts, emphasizing the importance of considering various treatment options and their implications for patients.

## Case presentation

A 14-year-old Iranian girl came to the outpatient clinic with a chief complaint of vague progressive pain in the left upper quadrant of the abdomen for 7 months. She also had suffered from left shoulder pain, fullness, early satiety, and shortness of breath for a month. The patient was in a completely healthy state until 7 months previously when she woke up with acute severe pain in her left shoulder. Exactly 3 days later, the hypochondrial pain was noticed. She hardly could breathe or lay down due to extreme aches. Primary workups revealed nothing except microcytic anemia [hemoglobin 10.3 g/dl, mean corpuscular volume (MCV) 63.6 fL]. Analgesics and folic acid were prescribed accordingly. The patient was asked for a follow-up visit a month later. Unfortunately, after three monthly outpatient visits, the pain progressed, and her physician requested no further diagnostic modalities. Due to ongoing pain and inadequate treatment, she came to our outpatient clinic for reevaluation. There was no nausea and vomiting, jaundice, fever, or bowel and urinary symptoms. She was living in the countryside and gave us a history of unwashed fruit ingestion. Examination revealed an asymmetric distended abdomen and a large palpable mass in the left hypochondrium, which was not tender. Vital signs were within normal limits. No past medical history was reported. Basic metabolic panel data was all in normal ranges.

**Fig. 1:**
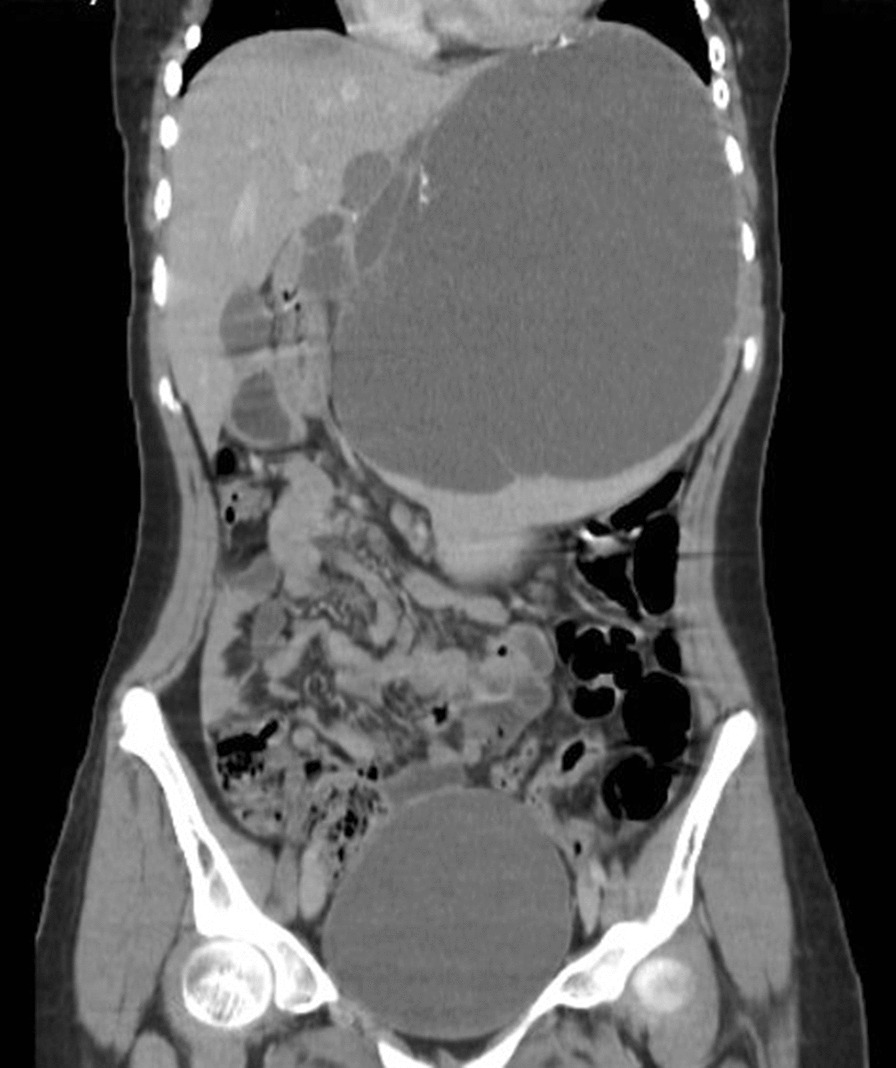
Computed tomography scan revealing a giant cyst in the spleen

Subsequently, US was requested, which showed a huge irregular wall cystic lesion below the left hemidiaphragm that pushed the hemidiaphragm upward, measuring about 220 mm × 150 mm × 160 mm with a few septa in it. Computed thermography (CT) revealed an enlarged spleen in size measuring 241 mm and intraparenchymal cystic lesion measuring 205 mm × 180 mm × 150 mm in the spleen containing thin septation and fine calcifications in-wall, suggestive of hydatid cyst versus epidermoid cyst (Fig. 1). Due to the compressive effect of the cyst, the left kidney was forced to the lower quadrant. Also, stomach, C loop of the duodenum, and pancreas had deviated to the right side. Liver, biliary tract, right kidney, and right and left adrenal glands were normal.

Due to the size of the cyst and its compressive effect, the patient was prepared to undergo total splenectomy with an impression of a hydatid cyst based on the endemicity of *Echinococcus granulosus* in the region. Albendazole was prescribed, 400 mg twice a day, and the patient was prepared for surgery.

**Fig. 2:**
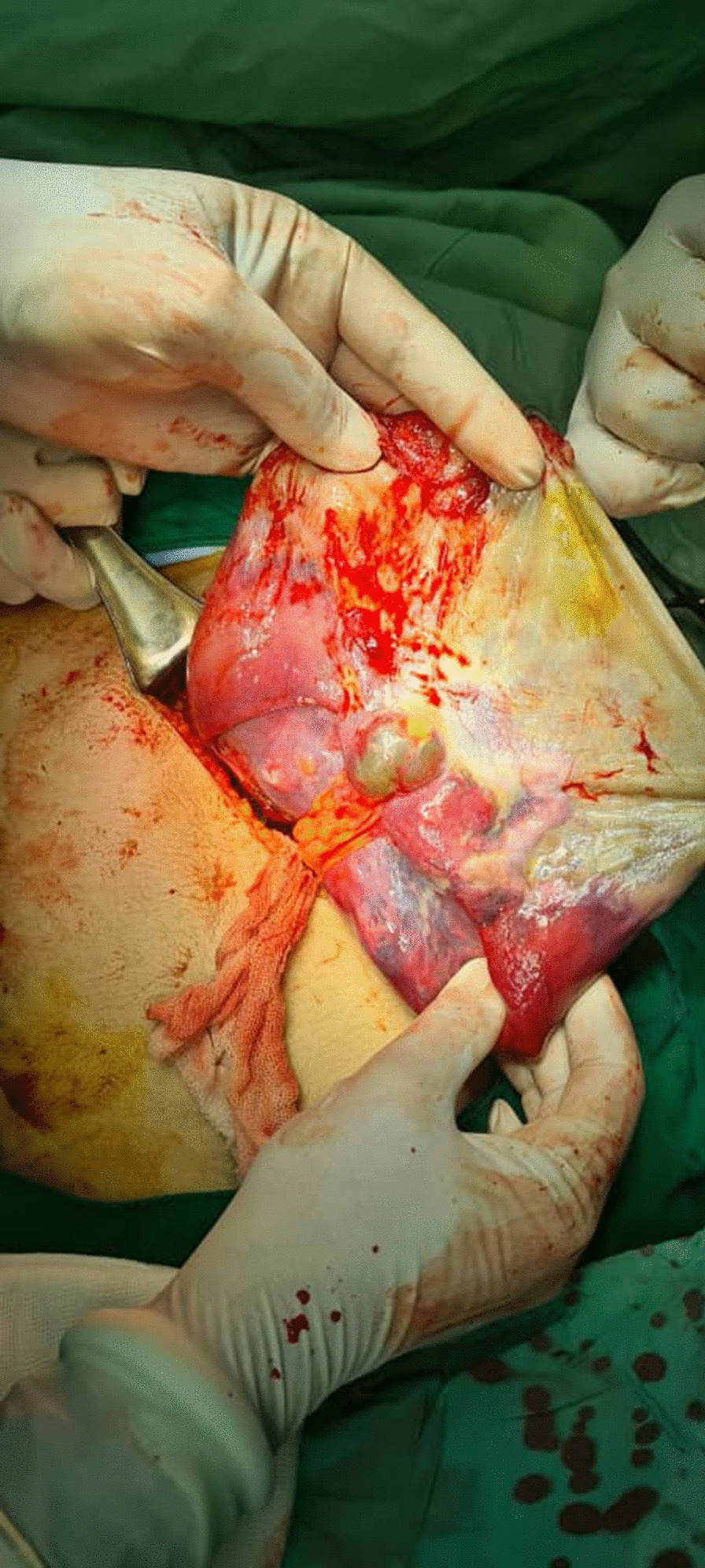
Intraoperative picture showing a drained cyst

In the operation room, a left subcostal incision in the fascia made an entrance to the peritoneal cavity. A huge splenic cyst was detected on first inspection. To facilitate the surgical procedure, enhance visualization, and minimize operative complications such as the accidental risk of rupture and anaphylaxis, the surgeon intentionally drained the cyst. Approximately 2 L of dark brown fluid were aspirated from the cyst. The cyst’s adhesions to adjacent organs such as the stomach, liver, and left side of the diaphragm were released. Splenocolic, splenophrenic, and splenorenal ligaments were dissected. Afterward, the short gastric artery was ligated, and the spleen was completely excised and was prepared for pathological examinations (Fig. 2).

Surprisingly, the pathologist has turned the suspected impression into a simple splenic cyst.

## Discussion

Simple cysts of the spleen are not common. The majority of the cases are classified as epithelial cysts. They are rare, including only around 10% of benign non-parasitic cysts. Most of them are asymptomatic, and they are accidental findings during abdominal US or CT scan [6]. Though, in recent years, the number of diagnosed simple splenic cysts seems to have increased because of the extended use of abdominal imaging modalities [7]. These cysts may predispose the patient to splenic rupture in cases of increased intraabdominal pressure or blunt abdominal trauma [8].

Considering the rarity of the disease, there is no precise statistic on the proportion of each etiologies in splenic cysts in the literature. According to a review, more than 20 cases of hydatid splenic cyst were reported from the country in the span of 20 years [8]. Another 15-year study has reported 14 cases of splenic hydatid cyst in southern Iran [9]. Overall, Iran is an endemic area for echinococcosis and the prevalence is relatively high [10]. On the other hand, non-parasitic cysts were only reported in a few studies; however, due to the asymptomatic nature of the disease, data could be unreliable [11–13].

A variety of reports in the medical literature have documented cases of huge splenic cysts. To the best of our knowledge, in terms of spleen size, we found a primary non-parasitic splenic cyst reported in a 17-year-old female, which surpassed the size of our case at 320 mm × 210 mm × 156 mm. Corresponding to our case, the patient’s primary complaint was pain in the left upper quadrant of the abdomen. Similar diagnostic approaches were employed, with confirmation of the splenic cystic mass through US and abdominal CT. Given the risks associated with recurrence, spontaneous or traumatic rupture, limited healthy splenic parenchyma, and involvement of the splenic hilar vessels, a radical splenectomy was deemed necessary and performed [14]. Another comparable case documented in the literature involved a 10-year-old Yemeni boy with an enlarged spleen measuring 200 mm × 160 mm × 180 mm. The patient exhibited symptoms akin to those in our case. Given the endemicity of hydatid cysts in Yemen, physicians adopted a similar diagnostic and treatment approach. However, in contrast to our case, pathological examinations confirmed cystic hydatidosis, aligning with their expectations [15, 16]. In another instance involving a massive non-parasitic mesothelial splenic cyst measuring 180 mm × 140 mm × 100 mm, the initial attempt to de-roof the cyst resulted in complications from secondary infection. Subsequently, the patient underwent an additional operation, during which a partial splenectomy was successfully performed. The outcome of this procedure was favorable, and the patient experienced no complications during the 6-month follow-up period [17]. Another case involved a non-parasitic huge splenic cyst measuring 170 mm × 140 mm × 140 mm, where dome resection was safely performed using indocyanine green fluorescence and percutaneous needle grasper. This approach effectively preserved the splenic immunological function [18].

The symptoms are associated with the size of the cyst and its pressure on adjacent structures. Complications such as infection, splenic cyst rupture, and/or bleeding can lead to life-threatening intraabdominal hemorrhage. Patients with large splenic cysts may have early satiety and left upper quadrant abdominal discomfort or pain. In some cases, the effect on the cardiorespiratory system may induce pleuritic pain, dyspnea, and persistent cough. Also, large cysts may lead to splenomegaly, which causes shoulder pain, nausea, vomiting, and constipation [3, 8, 11]. In addition, disseminated abdominal hydatidosis is uncommon. It might be due to peritoneal implantation occurring as a result of traumatic or surgical rupture of the cyst. Besides, primary dissemination is rare and accounts for 2% of intraabdominal hydatidosis, which can be diagnosed by ultrasound or CT scan [19].

Laparotomy with splenectomy has been the preferred method for the treatment of primary splenic cysts. Today, more conservative surgical procedures have been recommended, especially in children and young adults, to avoid overwhelming post-splenectomy infection [20]. Injection of sclerosing agents or percutaneous drainage has been used in limited cases as a more conservative alternative for small cysts, but these procedures can be associated with high recurrence rates [21, 22]. Besides, conservative management is recommended for asymptomatic patients with small cysts and where imaging features are compatible with a non-parasitic origin [23]. However, complete splenectomy is still reserved for cases where cyst excision cannot be done otherwise [2]. Therefore, we have selected the total splenectomy considering the presumed nature of the cyst and its huge size and location, which was in in splenic hilum. Moreover, due the size of the cyst and its compressive effect, laparoscopic approaches with lesser complications would not be achieved.

In such these cases, the preoperative differentiation between parasitic and non-parasitic is vital due to their variations in diagnosis and treatment. Parasitic splenic cysts or splenic hydatid disease must be excluded before invasive procedure due to spillage of the cystic contents, which may lead to anaphylactic shock or intraperitoneal dissemination of *Echinococcus* species [4, 24]. Accordingly, surgeons should keep in mind the possibility of a parasitic cyst when no reliable diagnosis has been made. Benzimidazole therapy is not necessary for treating splenic hydatidosis, although it is essential to perform splenectomy without rupturing and spilling the cysts [12].

Unfortunately, hydatid serology is not completely reliable, with reported sensitivities ranging between 24% and 96%, depending on the site of disease and the method used. Additionally, imaging features consistent with parasitic cysts, including the presence of daughter cysts within the cyst cavity, calcification of the cyst wall, or concomitant cystic disease in other organs, are not always present. A definitive preoperative diagnosis is often impossible, and in such cases, an intra- or postoperative diagnosis may be unavoidable [5]. Also, sonographic and CT findings of splenic hydatidosis are not specific, and other splenic cystic lesions, such as an epidermoid cyst, splenic abscess, a pseudocyst, or a cystic neoplasm of the spleen, may present with a similar appearance. The history greatly helps the differential diagnosis, the presence of calcification in the cyst wall, the presence of daughter cysts in a large cystic lesion, or concomitant cystic lesions in the liver or other organs. In patients with negative serology and indeterminant ultrasound and CT examinations, it may be helpful to use MRI [25, 26]. Therefore, a thorough history is essential, and potential parasitic exposure in endemic countries or agricultural areas may help determine the cyst’s etiology and help reduce the number of unnecessary tests and diagnostic hypotheses [3, 5, 11]. Altogether, clinicians could not be assured of the diagnosis up to histopathologic examination. In this case, the patient’s suspicious history of unhygienic ingestion of fruits and vegetables and the endemicity of the area were all weighted for hydatid splenic cyst, but the pathologist has ruled out this entity.

## Conclusion

Simple cysts of the spleen are not common, additional attention should be paid while making differential diagnosis for any long-lasting vague non-specific abdominal pain. Early ultrasonographic studies and reevaluating the primary diagnosis may be the best option for non-responding and/or progressive pains to catch the cysts in smaller sizes. Considering this entity in addition to splenic hydatidosis, especially in an endemic area, for echinococcosis can be challenging, although paraclinical evaluations as well as a thorough history and potential parasitic exposure in endemic countries or agricultural areas may help. Total splenectomy seems to the only option for these huge splenic cysts; however, choosing the correct operational approach should be determined case by case. These lessons collectively stress the value of a comprehensive approach, enhancing diagnostic accuracy and optimizing patient care in managing splenic cysts in uncommon cases.

## Data Availability

All data generated during this study are included in this published article and its supplementary information files.
